# The Effect of a Class IV Therapeutic LASER on Post-Surgical Wound Healing Processes in *Canis familiaris* and *Felis catus*: A Preliminary Study

**DOI:** 10.3390/ani15142133

**Published:** 2025-07-18

**Authors:** Ana Lopes, Pedro Azevedo, L. Miguel Carreira

**Affiliations:** 1 Anjos of Assis Veterinary Medicine Centre (CMVAA), 2830-077 Barreiro, Portugal; pedro.almeida.azevedo@gmail.com; 2 Faculty of American LASER Study Club, ALSC, Altamonte Springs, FL 32714, USA; 3 Department of Clinics, Surgery, Faculty of Veterinary Medicine, University of Lisbon (FMV/ ULisboa), 1300-477 Lisbon, Portugal; 4 Interdisciplinary Centre for Research in Animal Health (CIISA), Faculty of Veterinary Medicine, University of Lisbon (FMV/ ULisboa), 1300-477 Lisbon, Portugal; 5 Associate Laboratory for Animal and Veterinary Sciences (AL4AnimalS), Vila Real, Portugal

**Keywords:** dog, cat, surgical wound, class IV therapeutic laser, wound healing

## Abstract

Class IV laser therapy has emerged as a promising non-invasive tool to promote tissue repair, but its effects on post-surgical wound healing in small animals remain underexplored. This preliminary study investigated the impact of class IV laser therapy on surgical wound healing in 49 dogs and cats. Each surgical incision was divided into a Laser Zone (LZ) and a Control Zone (CZ). Wound healing was assessed at three timepoints (T0, T1, T2) using standardized clinical parameters: skin thickness, skin color, presence of hematoma, regional temperature, skin elasticity, and presence of fluids. The LZ consistently showed reduced skin thickness, more vivid pinkish skin color, faster hematoma resolution, increased regional temperature, improved skin elasticity, and decreased fluid accumulation compared to the CZ. These effects were observed across different species, ages, sexes, and body condition scores, indicating robust biological consistency. The results suggest that class IV laser therapy accelerates post-surgical wound healing by modulating inflammation, enhancing vascularization, and supporting extracellular matrix remodeling. This technique may serve as a valuable adjunct to conventional wound management in veterinary practice. Further studies are needed to confirm these findings and explore long-term outcomes.

## Simple Summary

Post-surgical wound healing is a critical aspect of veterinary patient recovery and comfort. This study evaluated the clinical effect of class IV laser therapy on wound healing in dogs and cats after surgery. Over an eight-day period, laser therapy led to reduced skin thickness, more vivid pinkish skin color, faster resolution of hematomas, increased regional temperature, improved skin elasticity, and decreased fluid accumulation. These findings suggest that class IV laser therapy enhances tissue repair, vascularization, and local recovery mechanisms. The effects were consistent across species, ages, sexes, and body condition, indicating that this non-invasive tool may help improve healing and animal comfort in routine veterinary practice.

## 1. Introduction

The skin is the largest organ of mammals, accounting for about 15% of body weight and serving critical roles in homeostasis, protection, thermoregulation, sensory perception, and immune defense [1,2]. Its multilayered structure—epidermis, dermis, and hypodermis—provides a robust barrier while enabling regeneration after injury [3,4]. In veterinary medicine, efficient wound healing is essential for post-surgical recovery, minimizing infection risks, and improving patient comfort and prognosis [5].

Wound healing is a dynamic process with three overlapping phases: inflammation, proliferation, and remodeling [5,6]. Each stage is coordinated through interactions between inflammatory cells, fibroblasts, endothelial cells, cytokines, and extracellular matrix (ECM) components [7,8]. Factors such as tissue oxygenation, vascularization, pH, comorbidities, and local immune status influence the wound healing process [9,10,11]. Notably, maintaining a slightly acidic skin pH helps to prevent pathogen colonization and supports enzyme activity essential for epidermal renewal [12].

In recent years, photobiomodulation therapy (PBMT), particularly using class IV therapeutic lasers, has gained recognition as a promising tool in regenerative medicine. Class IV lasers emit high-power infrared light (typically in the 780–980 nm range), allowing deeper tissue penetration and direct stimulation of mitochondrial cytochrome C oxidase. This mechanism enhances ATP production, promotes cellular proliferation, stimulates collagen synthesis, and induces angiogenesis [5,13,14]. These effects contribute to all stages of wound healing: they reduce inflammation and oxidative stress during the inflammatory phase, support fibroblast and keratinocyte activity during the proliferative phase, and facilitate organized ECM remodeling during the final maturation phase [6,15,16,17].

Although the literature on PBMT has grown steadily in both human and veterinary contexts, controlled studies specifically investigating its application in companion animals—especially cats—remain limited. Furthermore, there is a lack of intra-individual controlled studies that evaluate differential healing responses within the same animal and in the same surgical wound [7,9,10,11].

Therefore, the present study aimed to evaluate the effect of class IV laser therapy on the healing of surgical wounds in dogs (*Canis familiaris)* and cats (*Felis catus)*. Using an intra-individual split-wound design, this preliminary study investigates differences in key healing parameters—such as skin thickness, skin color, presence of hematoma, regional temperature, skin elasticity, and presence of fluids—over an 8-day postoperative period. This work contributes to the growing evidence base for PBMT in small animal practice and may support the development of standardized protocols for its clinical use.

## 2. Materials and Methods

### 2.1. Study Design and Animal Selection

This prospective intra-individual controlled study included a total of 49 animals (*n* = 49), comprising 25 dogs (*Canis familiaris)* and 24 cats (*Felis catus*) of both sexes (27 females and 22 males), various ages, and breeds. All patients underwent soft tissue and orthopedic procedures performed under aseptic conditions using a CO_2_ surgical laser technique, ensuring precise tissue incision, reduced intraoperative bleeding, and minimal collateral tissue trauma.

Each surgical incision was systematically divided into two equal zones: a cranial/proximal Laser-treated Zone (LZ) and a caudal/distal Control Zone (CZ) without therapeutic laser application. The cranial/proximal segment was consistently designated for laser therapy. This standardized approach accounted for anatomical and physiological factors (e.g., typical blood flow direction) and practical considerations, such as patient positioning and consistent reproducibility, across cases. (Note: for vertical or limb incisions, the terms proximal/distal are used instead of cranial/caudal to maintain anatomical accuracy).

To ensure the feasibility of the split-wound approach and meaningful intra-individual comparison, only surgical incisions measuring at least 5 cm in length were included, providing a minimum of 2.5 cm for each zone (LZ and CZ). The depth and anatomical location of the incisions varied, depending on the specific surgical indication; however, the intra-individual design ensured that each patient served as its own control, effectively minimizing the impact of local tissue variability [7,9,18].

Animals with any current or previous oncological disease, metabolic or endocrine disorders (e.g., diabetes mellitus, hypothyroidism, Cushing’s syndrome), or any other systemic condition that could affect wound healing were excluded. Cases where complete postoperative follow-up was not feasible were also excluded.

Written informed consent was obtained from all animal owners. The study protocol was approved by the institutional ethics committee (Ref. 015/2022).

### 2.2. Laser Treatment Protocol

Laser therapy was performed using a class IV diode laser system (Doctor Vet Therapy Laser, LAMBDA S.p.A., Vicenza, Italy) emitting a combination of wavelengths (660, 808, and 915 nm) in continuous wave (CW) and pulse modes. Irradiation was applied immediately postoperatively only once. The energy dose was adjusted according to the estimated wound area as follows:

▪ Post-op S: 5 cm^2^ area; 25 s; 2 W output; total energy 50 J; dose per area: 10 J/cm^2^.
▪ Post-op M: 25 cm^2^ area; 2 min 5 s; 2 W output; total energy 250 J; dose per area: 10 J/cm^2^.
▪ Post-op L: 50 cm^2^ area; 4 min 10 s; 2 W output; total energy 500 J; dose per area: 10 J/cm^2^.

Frequencies used included CW, 1, 2, 10, and 25 kHz, with distinct purposes [17,19] as follows:

▪ 1 kHz: epithelialization;
▪ 2 kHz: fibroblast stimulation;
▪ 10 kHz: infection control;
▪ 25 kHz: antimicrobial effect.

These frequency settings are based on previous validated protocols [10,12]. The laser beam was applied at least two passes to ensure homogeneous energy distribution over the treatment area.

### 2.3. Evaluation Parameters and Timeline

The surgical incision of each patient was divided into two equal parts as follows:

▪ Laser Zone (LZ): cranial/proximal segment receiving class IV laser application;
▪ Control Zone (CZ): caudal/distal segment without laser application.

Three postoperative timepoints were used for evaluation as follows:

▪ T0: immediately after surgery;
▪ T1: 48 h post-surgery;
▪ T2: 8 days post-surgery.

Wound healing was assessed using a validated scoring system adapted from Vitor & Carreira (2015) [18], including the following clinical parameters: skin thickness, skin color, presence of hematoma, regional temperature, skin elasticity, and presence of fluids. Regional temperature was measured using a non-contact infrared thermometer. To enhance reproducibility and minimize observer bias, standardized photographic monitoring was performed at each evaluation point under controlled lighting, distance, and scale. All assessments were performed by the same investigator to minimize variability and ensure consistency.

### 2.4. Statistical Analysis

Data was recorded in Microsoft Excel and analysed using IBM SPSS Statistics version 29 (Windows). Descriptive statistics included means, standard deviations, and frequencies. The Shapiro–Wilk test was used to check normality, and Levene’s test was used for homogeneity of variance. Inferential statistics comprised the following:

▪ Repeated measures ANOVA for intra-group comparisons over time (normally distributed data);
▪ Student’s *t*-test for independent samples where assumptions of normality and homogeneity were met;
▪ Mann–Whitney U test for non-normally distributed data;
▪ Fisher’s exact test for categorical associations with low-frequency outcomes;
▪ Cochran’s Q test for repeated binary variables across timepoints.

Categorical outcomes were coded as binary (dummy) variables. A *p*-value ≤ 0.05 was considered statistically significant.

## 3. Results

### 3.1. Sample Characterization

The total sample consisted of 49 animals, of which 51% (*n* = 25) were *Canis familiaris* (dogs) and 49% (*n* = 24) were *Felis catus* (cats). Female animals represented 55.1% of the total sample. Most of the animals were neutered (67.3%), with a higher prevalence in cats (83.3%) compared to dogs (52.0%). The mean age was 6.90 ± 4.30 years across all animals, 6.82 ± 4.64 years in dogs, and 7.13 ± 4.18 years in cats. Regarding body weight, dogs showed a higher average (17.50 ± 12.57 kg) than cats (4.27 ± 1.20 kg). The most frequent body condition score, based on the nine-point LaFlamme scale, was 6 in both species. Full descriptive data is presented in Table 1.

### 3.2. Wound Healing Parameters

All parameters were assessed at three timepoints: T0 (immediate post-surgery), T1 CZ (48 h post-surgery in the Control Zone), T1 LZ (48 h post-surgery in the Laser Zone), T2 CZ (8 days post-surgery in the Control Zone), and T2 LZ (8 days post-surgery in the Laser Zone). The analysis was performed on the total sample and separately for dogs and cats.

#### 3.2.1. Skin Thickness

**Figure 1:**
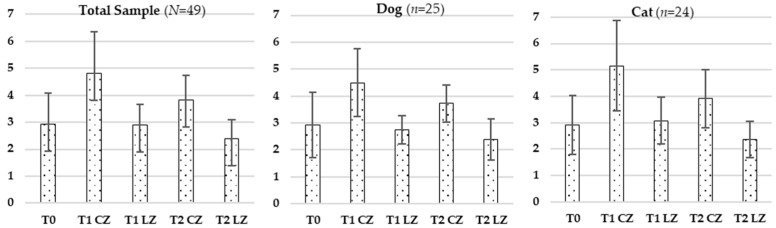
Evolution of skin thickness in the total sample (*N* = 49), dogs (*n* = 25), and cats (*n* = 24) across different evaluation timepoints (T0, T1, T2) considering the Laser Zone (LZ) and the Control Zone (CZ).

Statistically significant differences in skin thickness were observed across all timepoints in the total sample (F (4.192) = 80.008; *p* < 0.001), in dogs (F (4.21) = 17.756; *p* < 0.001) and cats (F (4.20) = 37.707; *p* < 0.001), as analyzed by repeated measures ANOVA. Post-op comparisons (Cochran’s test) revealed multiple significant differences between treatment conditions and timepoints. Full data are summarized in Table 2 and Figure 1.

#### 3.2.2. Skin Color

**Figure 2:**
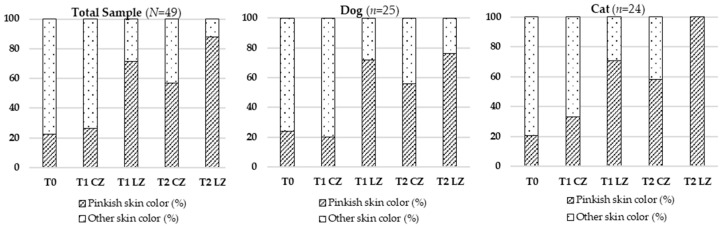
Evolution of skin color in the total sample (*N* = 49), dogs (*n* = 25), and cats (*n* = 24) across different evaluation timepoints (T0, T1, T2) considering the Laser Zone (LZ) and the Control Zone (CZ).

Significant changes in skin color were also detected (Cochran’s Q test), both in the total sample (χ^2^ (4) = 59.535; *p* < 0.001) and by species. Improved normalization of skin color (pinkish hue) was notably more frequent at T2 LZ. The detailed results are illustrated in Table 3 and Figure 2.

#### 3.2.3. Presence of Hematoma

**Figure 3:**
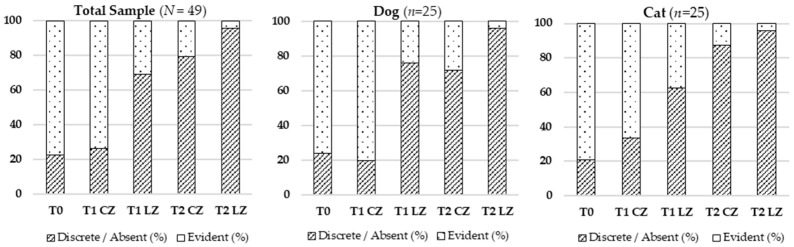
Evolution of the presence of hematoma in the total sample (*N* = 49), dogs (*n* = 25), and cats (*n* = 24) across different evaluation timepoints (T0, T1, T2) considering the Laser Zone (LZ) and the Control Zone (CZ).

There was a significant reduction in hematoma presence across timepoints in the total sample (χ^2^ (4) = 77.353; *p* < 0.001), dogs (χ^2^ (4) = 41.941; *p* < 0.001), and cats (χ^2^ (4) = 38.038; *p* < 0.001). The most marked reduction was seen at T2 LZ. See Table 4 and Figure 3 for visualization.

#### 3.2.4. Regional Temperature

**Figure 4:**
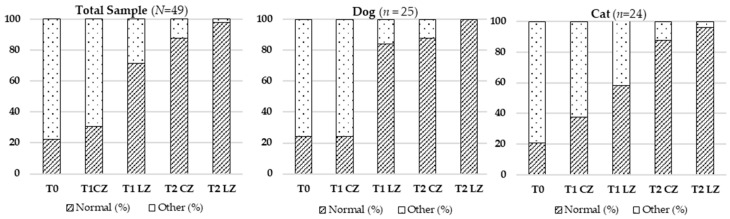
Evolution of regional temperature in the total sample (*N* = 49), dogs (*n* = 25), and cats (*n* = 24) across different evaluation timepoints (T0, T1, T2) considering the Laser Zone (LZ) and the Control Zone (CZ).

Significant variations in regional temperature were observed across timepoints in the total sample (χ^2^ (4) = 86.188; *p* < 0.001), dogs (χ^2^ (4) = 53.438; *p* < 0.001), and cats (χ^2^ (4) = 36.750; *p* < 0.001), with normalization more prominent at T2 LZ. Refer to Table 5 and Figure 4.

#### 3.2.5. Skin Elasticity

**Figure 5:**
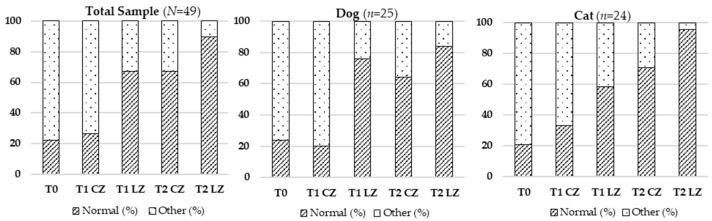
Evolution of skin elasticity in the total sample (*N* = 49), dogs (*n* = 25), and cats (*n* = 24) across different evaluation timepoints (T0, T1, T2) considering the Laser Zone (LZ) and the Control Zone (CZ).

Skin elasticity improved significantly over time in all groups, particularly after laser application (LZ). These changes were confirmed by Cochran’s Q test (total sample: χ^2^ (4) = 32.046; *p* < 0.001). The complete results are shown in Table 6 and Figure 5.

#### 3.2.6. Presence of Fluids

**Figure 6:**
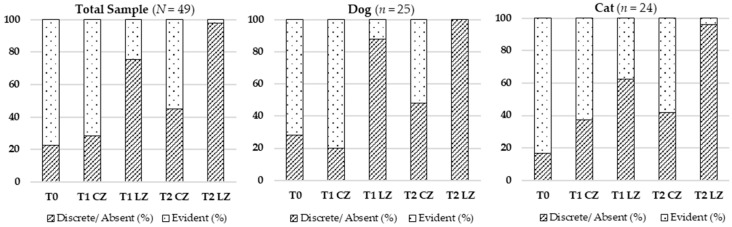
Evolution of the presence of fluids in the total sample (*N* = 49), dogs (*n* = 25), and cats (*n* = 24) across different evaluation timepoints (T0, T1, T2) considering the Laser Zone (LZ) and the Control Zone (CZ).

There was a statistically significant reduction in fluid presence in all groups over time (total sample: χ^2^ (4) = 73.508; *p* < 0.001). Near-complete resolution was observed at T2 LZ. See Table 7 and Figure 6 for graphical data.

### 3.3. Inter-Species Comparison (Dogs vs. Cats)

**Figure 7:**
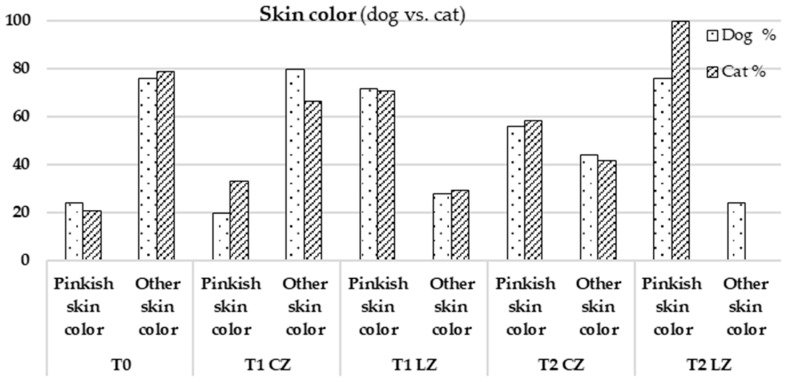
Comparison between dogs (*Canis familiaris*, *n* = 25) and cats (*Felis catus*, *n* = 24) regarding the parameter skin color.

No significant differences were found in most parameters between dogs and cats, except for higher pinkish skin color in cats at T2 LZ (*p*-value = 0.022) [20]. The results are presented in Figure 7.

### 3.4. Influence of Age, Sex, and Body Condition Score

No age, sex, or body condition score differences were found for skin thickness, regional temperature, skin elasticity, or presence of fluids.

Logistic regression indicated that sex was a significant predictor for pinkish skin color in the CZ (*p* = 0.014), with females more likely to present pinkish skin color. No significant predictors were identified for the LZ (Table 8) [8,21].

In the CZ, body condition score was a significant predictor (*p* = 0.025), with worse condition associated with fewer evident hematomas. No significant effects were found in the LZ. The results are detailed in Table 9 [15,21].

## 4. Discussion

This study evaluated the therapeutic effects of class IV laser therapy on the healing process of post-surgical wounds in dogs and cats. The sample included 49 animals of different ages, sexes, body weights, and body condition scores, allowing for a broad assessment of the clinical effects of photobiomodulation (PBMT) using a class IV therapeutic laser.

The intra-individual split-wound design isolated the independent variable (laser treatment) while keeping other intrinsic and extrinsic variables constant. This approach minimized confounding factors, reduced bias, and improved internal validity and statistical robustness, consistent with other studies supporting intra-individual models as the gold standard for clinical comparisons [1,2,3,4,7,16,17,22].

Skin thickness decreased significantly in the Laser Zones (LZs) compared to Control Zones (CZs) at all timepoints, suggesting reduced local inflammation and lower extracellular matrix (ECM) density. During the inflammatory phase, vasodilation and increased vascular permeability enable immune cell infiltration and protein extravasation, leading to localized swelling. Transition to the proliferative phase involves fibroblast proliferation, ECM remodeling, and granulation tissue formation. Class IV laser therapy accelerates this process by modulating pro-inflammatory cytokines (e.g., TNF-α, IL-1β) and enhancing anti-inflammatory mediators, like IL-10 [5,18]. It also stimulates cytochrome C oxidase in mitochondria, boosting ATP production and cellular metabolism. This bioenergetic effect promotes fibroblast activity, type III collagen synthesis, and myofibroblast differentiation, supporting wound contraction and tissue remodeling [5,6]. Growth factors such as TGF-β, FGF, and IGF are upregulated in response to laser exposure, contributing to the observed reduction in skin thickness as an indicator of faster healing.

No significant differences in skin thickness were observed between dogs and cats or across different ages and sexes, suggesting that the laser consistently modulated healing mechanisms regardless of physiological profile. Estrogens typically promote tissue regeneration by enhancing epidermal thickness, vascularization, and collagen deposition, whereas androgens can counteract these effects [8,21]. The consistent outcomes here indicate that the laser’s effects may override baseline hormonal differences.

Skin color, an indicator of vascular perfusion and oxygenation, appeared as a more vivid pinkish hue in Lazer Zones (LZs), especially at T2, aligning with the peak of the proliferative phase characterized by angiogenesis driven by VEGF, FGF, and TGF-β [9,23]. The laser likely promoted VEGF expression through increased ATP production and mitochondrial activation, resulting in improved microvascular density and tissue oxygenation. This effect was more evident in cats, possibly due to their thinner epidermis, reduced subcutaneous fat, and more superficial vasculature, enhancing light absorption [10].

In Control Zones (CZs), females showed more pronounced pinkish skin color than males, likely due to estrogen-mediated vasodilation and angiogenesis [8]. However, this difference was not evident in Laser Zones (LZs), suggesting that the laser-induced vascular response compensated for hormonal variations. These hypotheses are supported by evidence on ROS and mitochondrial pathways involved in VEGF upregulation and vascular remodeling [10,16,17,19].

Hematoma resolution occurred faster in LZs, reflecting improved inflammation control and vascular repair. Class IV laser therapy is thought to enhance lymphatic drainage, nitric oxide release, and endothelial stabilization [11,13,17,19,23]. ROS signalling and cytochrome C oxidase activation reduce capillary permeability and stimulate macrophage activity, facilitating erythrocyte phagocytosis and the degradation of extravascular hemoglobin [4,6,13,17,19].

It is important to note that lymphatic flow is inherently continuous across the wound area, even when divided anatomically into treated and Control Zones. This means that drainage in one segment may partially influence the adjacent zone—a recognized limitation of the split wound model. Nevertheless, the localized bio-stimulatory effect of laser therapy can modulate lymphatic activity within the treated zone (LZ) and promote more efficient clearance of interstitial fluids.

Additionally, animals with higher body condition scores showed prolonged hematoma presence in CZs, likely because excess subcutaneous fat can compress lymphatic vessels, reduce drainage efficiency, and contribute to low-grade inflammation. This mechanism explains why fluid and hematoma persistence were more evident in the CZs of overweight animals. In contrast, the laser’s stimulation of endothelial stability and lymphatic flow appeared to offset this disadvantage, resulting in faster resolution of hematoma in LZs.

Skin temperature increased significantly over time in LZs, consistent with metabolic activation induced by laser therapy. Enhanced mitochondrial function increases ATP production, vasodilation, and local perfusion—key factors in active tissue repair [4,6,14]. This effect was uniform across species and unaffected by sex, age, or body condition score.

Skin elasticity, reflecting ECM integrity, improved significantly in LZs. This improvement was likely due to increased synthesis of collagen types I and III, elastin, and fibronectin driven by elevated ATP levels and fibroblast activity [15,17,19]. Laser therapy also modulates MMPs and TIMPs, ensuring balanced ECM turnover [14,16]. While sex hormones can affect dermal elasticity [8,21], the laser’s bio-stimulating effect appeared to offset these variations, resulting in more uniform tissue quality.

The presence of fluids, including lymphatic and serosanguinous exudate, decreased more rapidly in LZs. This outcome can be explained by enhanced endothelial and lymphatic function via cytochrome C oxidase activation and improved ATP-driven ion transport [4,6,17,24]. Aquaporin regulation and reduced histamine-mediated capillary permeability [12,15,23] also contributed to faster fluid resolution. Again, the continuous nature of lymphatic circulation across the wound zones must be acknowledged as a possible confounding factor when interpreting intra-wound comparisons.

Given the preliminary nature of this study, several limitations should be acknowledged. First, the follow-up period was relatively short and focused on early healing phases, limiting conclusions about long-term outcomes. Second, although standardized photographic monitoring was implemented, advanced imaging methods such as elastography, digital thermography, or histological analysis were not performed. Third, the absence of blinded outcome assessors could result in observer bias. Fourth, despite excluding animals with obvious oncological history or severe systemic disease, undetected comorbidities, like diabetes or hypothyroidism, may have influenced healing responses. Fifth, only a single laser application was used immediately post-surgery, which does not fully represent multi-session protocols common in clinical practice. Finally, the inclusion of various surgical procedures—both soft tissue and orthopedic—performed with the CO_2_ laser technique naturally introduced variability in wound depth, anatomical location, and healing characteristics.

## 5. Conclusions

This study provides preliminary evidence that class IV laser therapy can accelerate post-surgical wound healing in dogs and cats by promoting better inflammation control, tissue remodeling, and vascularization. The intra-individual design minimized confounding factors and supported the consistent effects observed. Class IV laser therapy shows promise as a non-invasive adjunct in routine veterinary wound care. Further research should confirm these findings and assess long-term outcomes.

## Figures and Tables

**Table 1:** Characterization of the total sample (*N* = 49) and the two species groups considered in the study—dogs (*Canis familiaris*, *n* = 25) and cats (*Felis catus*, *n* = 24)—regarding age, body weight, sex, body condition score, and reproductive status.

Parameters	Total Sample	Dog	Cat
Age (years)	6.90 ± 4.3%	6.82 ± 4.6%	7.13 ± 4.2%
Body weight (Kg)	12.00 ± 11.6%	17.50 ± 12.6%	4.27 ± 1.2%
Sex (female)	27 (55.1%)	15 (60.0%)	12 (50.0%)
Sex (male)	22 (44.9%)	10 (40.0%)	12 (50.0%)
Body condition score (1–9)	1: 2 ± 4.7%	1: 1 ± 4.5%	1: 1 ± 4.8%
	2: 4 ± 9.3%	2: 2 ± 9.1%	2: 2 ± 9.5%
	3: 3 ± 7.0%	3: 1 ± 4.5%	3: 2 ± 9.5%
	4: 7 ± 16.3%	4: 5 ± 22.7%	4: 2 ± 9.5%
	5: 10 ± 23.3%	5: 5 ± 22.7%	5: 5 ± 23.8%
	6: 11 ± 25.6%	6: 6 ± 27.3%	6: 5 ± 23.8%
	7: 4 ± 9.3%	7: 1 ± 4.5%	7: 6 ± 27.3%
	8: 2 ± 4.7%	8: 1 ± 4.5%	8: 6 ± 27.3%
Reproductive status (neutered)	33 (67.3%)	13 (52.0%)	20 (83.3%)
Reproductive status (intact)	16 (32.7%)	12 (48.0%)	4 (16.7%)

*N*—Sample size; *n*—subsample.

**Table 2:** Characterization of the total sample (*N* = 49) and the two species groups considered in the study—dogs (*Canis familiaris*, *n* = 25) and cats (*Felis catus*, *n* = 24)—regarding skin thickness.

Evaluation Moment	Total Sample	Dog	Cat
T0	2.93 ± 1.16%	2.92 ± 1.22%	2.92 ± 1.11%
T1 CZ	4.82 ± 1.52%	4.49 ± 1.26%	5.15 ± 1.71%
T1 LZ	2.91 ± 0.74%	2.75 ± 0.52%	3.07 ± 0.89%
T2 CZ	3.82 ± 0.91%	3.73 ± 0.69%	3.91 ± 1.10%
T2 LZ	2.38 ± 0.72%	2.38 ± 0.76%	2.36 ± 0.68%

**Table 3:** Characterization of the total sample (*N* = 49) and the two species groups considered in the study—dogs (*Canis familiaris*, *n* = 25) and cats (*Felis catus*, *n* = 24)—regarding skin color (% pinkish skin color / % other skin color).

Evaluation Moment	Total Sample	Dog	Cat
T0	22.4/77.6	24.0/76.0	20.8/79.2
T1 CZ	26.5/73.5	20.0/80.0	33.3/66.7
T1 LZ	71.4/28.6	72.0/28.0	70.8/29.2
T2 CZ	57.1/42.9	56.0/44.0	58.3/41.7
T2 LZ	87.8/12.2	76.0/24.0	100.0/0.0

**Table 4:** Characterization of the total sample (*N* = 49) and the two species groups considered in the study—dogs (*Canis familiaris*, *n* = 25) and cats (*Felis catus*, *n* = 24)—regarding the presence of hematoma (% discrete or absent/% evident).

Evaluation Moment	Total Sample	Dog	Cat
T0	22.4/77.6	24.0/76.0	20.8/79.2
T1 CZ	26.5/73.5	20.0/80.0	33.3/66.7
T1 LZ	69.4/30.6	76.0/24.0	62.5/37.5
T2 CZ	79.6/20.4	72.0/28.0	87.5/12.5
T2 LZ	92.9/4.1	96.0/4.0	95.8/4.2

**Table 5:** Characterization of the total sample (*N* = 49) and the two species groups considered in the study—dogs (*Canis familiaris*, *n* = 25) and cats (*Felis catus*, *n* = 24)—regarding regional temperature (% normal/% other).

Evaluation Moment	Total Sample	Dog	Cat
T0	22.5/77.6	24.0/76.0	20.8/79.2
T1 CZ	30.6/69.4	24.0/76.0	37.5/62.5
T1 LZ	71.4/28.6	84.0/16.0	58.3/41.8
T2 CZ	87.8/12.2	88.0/12.0	87.5/12.5
T2 LZ	98.0/2.0	100.0/0.0	95.8/4.2

**Table 6:** Characterization of the total sample (*N* = 49) and the two species groups considered in the study—dogs (*Canis familiaris*, *n* = 25) and cats (*Felis catus*, *n* = 24)—regarding skin elasticity (% normal/ % other).

Evaluation Moment	Total Sample	Dog	Cat
T0	22.5/77.6	24.0/76.0	20.8/79.2
T1 CZ	26.5/73.5	20.0/80.0	33.3/66.7
T1 LZ	67.4/32.7	76.0/24.0	58.3/41.7
T2 CZ	67.4/32.7	64.0/36.0	70.8/29.2
T2 LZ	89.8/10.2	84.0/16.0	95.8/4.2

**Table 7:** Characterization of the total sample (*N* = 49) and the two species groups considered in the study—dogs (*Canis familiaris*, *n* = 25) and cats (*Felis catus*, *n* = 24)—regarding the presence of fluids (% discrete or absent/ % evident).

Evaluation Moment	Total Sample	Dog	Cat
T0	22.5/77.6	28.0/72.0	16.7/83.3
T1 CZ	28.6/71.4	20.0/80.0	37.5/62.5
T1 LZ	75.5/24.5	88.0/12.0	62.5/37.5
T2 CZ	44.9/55.1	48.0/52.0	41.7/58.3
T2 LZ	98.0/2.0	100.0/0.0	95.8/4.2

**Table 8:** Logistic regression statistics illustrating the influence of age, gender, and body condition on the parameter skin color.

Parameters/Coefficient		B	SD	Wald	df	*p*-Value
	Gender	1.764	0.719	6.024	1	**0.014**
**CZ**	Body Condition	0.367	0.222	2.864	1	0.091
	Age	0.016	0.089	0.031	1	0.859
	Gender	0.600	0.985	0.371	1	0.543
**LZ**	Body Condition	−0.114	0.298	0.147	1	0.701
	Age	−0.091	0.124	0.541	1	0.462

**B**—Unstandardized coefficient; **SD**—standard deviation; **Wald**—statistical test B^2^/SD^2^; **df**—degree of freedom; ***p*-value**—statistical significance.

**Table 9:** Logistic regression statistics illustrating the influence of age, gender, and body condition on the parameter presence of hematomas.

Parameters/Coefficient		B	SD	Wald	df	*p*-Value
	Gender	−1.421	0.881	2.601	1	0.107
**CZ**	Body Condition	0.570	0.254	5.053	1	**0.025**
	Age	0.001	0.106	0,000	1	0.993
	Gender	−18.911	876.77	0.000	1	0.998
**LZ**	Body Condition	−0.040	0.477	0.007	1	0.933
	Age	0.032	0.210	0.023	1	0.880

**B**—Unstandardised coefficient; **SD**—standard deviation; **Wald**—statistical test B^2^/SD^2^; **df**—degree of freedom; ***p*-value**—statistical significance.

## Data Availability

The original contributions presented in the study are included in the article; further inquiries can be directed to the corresponding author/s.

## Abbreviations

The following abbreviations are used in this manuscript:

ECM	Extracellular Matrix
LZ	Laser Zone
CZ	Control Zone
ATP	Adenosine Triphosphate
ROS	Reactive Oxygen Species
NO	Nitric Oxide
VEGF	Vascular Endothelial Growth Factor
FGF	Fibroblast Growth Factor
TGF-β	Transforming Growth Factor Beta
IL-1β	Interleukin 1 Beta
IL-10	Interleukin 10
TNF-α	Tumor Necrosis Factor Alpha
MMP	Matrix Metalloproteinase
TIMP	Tissue Inhibitor of Metalloproteinase
T0, T1, T2	Timepoints: immediately post-op, 48 h, day 8
PBMT	Photobiomodulation Therapy
IGF	Insulin-like Growth Factor
